# Analysis of factors in community willingness to manage floods in East Java during the pandemic

**DOI:** 10.4102/jamba.v16i1.1598

**Published:** 2024-06-28

**Authors:** Eko Noerhayati, Soraya N. Mustika, Ita S. Ingsih, Anita Rahmawati

**Affiliations:** 1 Department of Civil Engineering, Faculty of Engineering, Universitas Islam Malang, Malang, Indonesia; 2 Department of Electronic, Faculty of Applied Science and Technology, Universitas Negeri Malang, Malang, Indonesia

**Keywords:** community willingness, environment, floods, manage, COVID-19, pandemic

## Abstract

Urbanisation is an important aspect of environmental management and requires special attention, as half of the world’s population lives in urban areas. As the urban population grows, this poses a number of challenges, particularly related to environmental issues and floods. Indonesia, a developing nation and the fourth most populous globally, with over 264 million inhabitants, faces significant environmental and flood concerns, notably exacerbated by the coronavirus disease 2019 (COVID-19) pandemic. These issues are poised to exert a profound and enduring influence on the populace. East Java province, one of the densely populated provinces in Indonesia, experiences complex environmental problems, floods, and air pollution. Therefore, this research identified the factors that influenced the community’s willingness to participate in environmental conservation and flood management in East Java province during the COVID-19 pandemic. This research employed a mixed-method approach, combined qualitative and quantitative methods, and utilised the triangular relationship theory. The result showed six predominant factors within the population of East Java that significantly affected the environmental conditions and the frequency of flood events in the area.

**Contribution:**

Floods in the city during the pandemic caused concern for those affected by the disaster and the implementation of activities adjusted government policies. For sustainability, the urban environment in Indonesia is working hard to anticipate flooding in cities. Apart from that, the government, private sector, community leaders, and the media also play an important role.

## Introduction

Approximately half the global population resides in urban areas, making urbanisation a pivotal focus of worldwide environmental management (Broere 2016; Chiang 2018; Mashiane et al. 2023; Musliu et al. 2023) that requires particular attention (Ahmed & Islam 2014). Historically, urban dwelling was far less prevalent, with less than 5% of the populace residing in cities during the 1800s. By the 1950s, 70% of the population of the world lived in rural areas (Ahmed & Islam 2014). The urban populace is currently on the rise, and mega-cities, such as those harbouring 10 million or more individuals, are projected to increase from slightly over 10 million in 1990 to a staggering 41 million by 2030 (Pouyat & Trammell 2019). Interestingly stated that a significant portion of population growth in developing nations stems from natural increases rather than rural-to-urban migration. In accordance with the 2020 National Census of Indonesia, it has been proven that the population of East Java province was 40.67 million in 2020 (Rohman & Wiyono 2022).

As urban populations continue to grow, various challenges emerge, particularly in environmental issues (Moore, Gould & Keary 2003; Panel UNEPIR 2011), specifically in developing countries. The same challenges are faced by South Asia and sub-Saharan African countries, where the increase in urban population has positive and negative impacts on human wellbeing and the environment (Kookana et al. 2020). Urban areas commonly grapple with issues such as floods, air pollution, waste management and access to clean water (Moore et al. 2003). In this study, we thoroughly examine the intricate relationship between community willingness to participate in environmental conservation and flood management, taking into account the unique challenges arising from the coronavirus disease 2019 (COVID-19) pandemic.

The world is currently under the strain of the novel COVID-19 pandemic. Indonesia (Uteulin & Zhientaev 2022), identified as a developing nation (Barakwan, Trihadiningrum & Bagastyo 2019; Busquet et al. 2019; Colenbrander et al. 2015), and standing as the fourth most populous country globally with over 264 million inhabitants (Sabani, Farah & Dewi 2019), is expected to experience substantial repercussions because of an extended duration of this crisis (Djalante et al. 2020). Within Indonesia, East Java province, a densely populated area, grapples with intricate environmental challenges, such as floods and air pollution (Suhardono et al. 2023; Tunas 2019; Tunas, Tanga & Oktavia 2020; Wardhani & Dugis 2020). Floods bring about significant adverse consequences in tropical areas, and the local population often struggles to mitigate their impacts (Rodysill et al. 2019). These environmental predicaments can be traced back to rapid population expansion, economic pressures, extensive land utilisation and the escalated use of motor vehicles, releasing pollutants into the atmosphere (Wardhani & Dugis 2020).

Several major cities in East Java, such as Surabaya, Sidoarjo and Malang face similar issues. Surabaya, a metropolitan city where most of its population consists of workers and migrants, experiences floods every time it rains (Fikri, Tjendani & Oetomo 2023; Hariyono et al. 2023; Imaduddina & Subagyo 2014; Imaaduddiin, Saud & Santoso 2022). Similarly, Sidoarjo, characterised by a high population density and a mix of small and large industries, shares this inundation challenge. Malang (Handayani & Prawito 2022; Hayati et al. 2022), renowned as an educational hub, has recently fallen prey to these regular flooding events, which were once infrequent (Hapsari et al. 2023; Hariyono & Kurniawan 2022; Utami & Bisri 2014). All these cities were interconnected by the Brantas River Basin, originating in Sumber Brantas Village, Bumiaji Sub-District, Batu City.

This river draws its waters from the reserves of Mount Arjuno, meandering through the areas of Malang, Blitar, Tulungagung, Kediri, Jombang and Mojokerto. Upon reaching Mojokerto Regency, the river bifurcates into the Kali Mas (flowing towards Surabaya) and the Kali Porong River (flowing towards Porong in Sidoarjo Regency). The Brantas River Basin encompasses a vast area of 11 800 km^2^, constituting one-quarter of the total area of East Java province. The primary river itself extends over a length of 320 km, winding its way around the active volcano Mount Kelud. This river basin system comprises three integral components, namely water sources, in-stream and out-of-stream requirements, as well as intermediary functions such as maintenance and recycling (Cai, Ringler & Rosegrant 2006).

Effective management of the Brantas River Basin is imperative to ensure a stable water supply, with a particular focus on the needs of Surabaya (Amalia & Soedjono 2019). The area experiences an average annual rainfall of 2000 mm, with approximately 85% occurring during the rainy season. This pattern often brings intense rainfall in short intervals, particularly affecting the aforementioned cities. The absence of adequate drainage systems further compounds the problem, frequently resulting in flooding. Furthermore, government authorities and the local community often allocate insufficient attention to environmental protection against the threat of floods. This issue necessitates year-round special consideration, specifically in countries with tropical climates. Similar concerns arise regarding air pollution, driven by heightened motor vehicle usage and industrial activities. In addition to the aforementioned factors, the conversion of land use without due regard for green open spaces poses another challenge. Many paddy fields are being transformed into residential areas to meet market demands and accommodate the increasing population. The year 2019 brought its challenges with the emergence of the coronavirus disease 2019, more commonly known as COVID-19 (Pasaribu et al. 2021).

**FIGURE 1:**
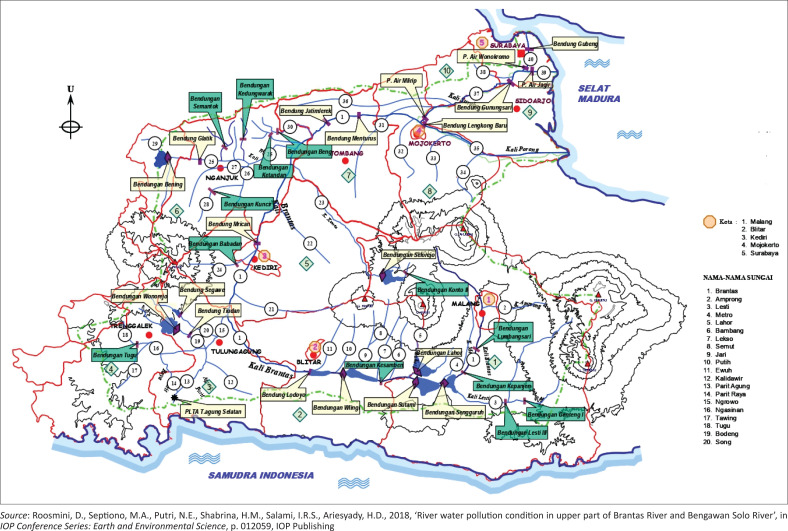
Brantas River basin map.

The imperative for community involvement in environmental conservation and flood management has gained prominence in recent years, reflecting a growing recognition of the pivotal role that local communities play in sustainable environmental practices. (Atanga 2020; Bark, Martin-Ortega & Waylen 2021). This river draws its waters from the reserves of Mount Arjuno, meandering through the areas of Malang, Blitar, Tulungagung, Kediri, Jombang, and Mojokerto. Upon reaching Mojokerto Regency, the river bifurcates into the Kali Mas (flowing towards Surabaya) and the Kali Porong River (flowing towards Porong in Sidoarjo Regency). Figure 1 is The Brantas River Basin encompasses a vast area of 11 800 km^2^, constituting one-quarter of the total area of East Java province. The primary river itself extends over a length of 320 km, winding its way around the active volcano Mount Kelud. This river basin system comprises three integral components, namely water sources, in-stream and out-of-stream requirements, as well as intermediary functions such as maintenance and recycling (Nabangchang et al. 2015).

## Literature review

Numerous studies have explored the inseparable connection between community risk perception and decision-making processes, contributing to the enhancement of adaptive actions in flood-prone communities (Dhar et al. 2023). High rainfall, drainage system blockages and/or failures, poor environmental conditions and inadequate land use planning are often cited as major contributors to flood-related issues. The substantial population growth in recent decades necessitates the development of extensive urban infrastructure. However, urbanisation processes have concurrently led to decreased soil permeability, resulting in floods and severe damages (Amaro et al. 2022). A significant portion of the population resides in flood-prone areas, either unaware of the flood issues or choosing these locations for their proximity to workplaces or affordability.

Policy-makers must implement development controls in floodplains, ensure better and regular waste collection methods, maintain proper drainage systems and establish effective flood defences (Srinivas, Singh & Shankar 2020). Microeconomic factors such as household costs and household member incomes can be collected and utilised for policy purposes (Nwosu, Anumudu & Nnamchi 2021). While many urban residents express a willingness to support sustainable urban drainage infrastructure development (Yang & Zhang 2021), floods persist, particularly during the rainy season. This situation mirrors the challenges faced in East Java province, Indonesia, where the recurring issue during every rainy season is flooding and waterlogging. This is primarily attributed to inadequate management and discharge of water (drainage), exacerbated by a lack of awareness and community participation in maintaining the drainage channels, leading to blockages by industrial and household waste.

The causes of floods can be both natural and anthropogenic. In urban areas, human activities resulting in spatial changes can influence natural alterations. Environmental degradation, such as the loss of ground cover in catchment areas (Obiahu & Elias 2020), river sedimentation causing riverbed shallowing, narrowing of river channels and more, can be attributed to human actions. Large-scale floods have undesirable impacts on physical, social, economic and environmental aspects. Flood mitigation requires community participation, as it is the community that can identify needs and prioritise them. They are most capable of articulating existing problems and taking responsive actions based on available local resources and capacities, enabling effective flood mitigation planning and implementation.

The Reasoned Action Theory (Nisson & Earl 2020) serves as the foundational model, supplemented by factors such as disaster concern, subjective norms and flood-related worries, all positively contributing to the intention to participate in community-based disaster preparedness schemes. The triangular relationship theory among citizens’ environmental concerns, media attention to environmental issues and policy outcomes holds significant implications for understanding environmental policy-making. Public opinion and media coverage mutually reinforce each other, but legislative action may negatively affect public opinion, while media attention may decrease once policy implementation occurs (Bakaki, Böhmelt & Ward 2020).

Studies suggest that raising awareness of sustainable environmental practices can lead to increased income acceptance and support for development, contributing to policymaking (Khan, Yu & Farooq 2023). The lack of knowledge about rainwater is a barrier to property development in communities, necessitating specific dissemination and informative activities (Maleki, Koohestani & Keshavarz 2020). The areas of flood risk management are effective communication to residents in flood-prone settlements, private sector concerns, community skills, and legal regulations for policy makers. The community is willing to invest in flood mitigation actions and flood insurance (Mai et al. 2020).

Therefore, the triangular relationship theory proposed by Zorzeta Bakaki can serve as the basis and be developed into a model of community willingness to care for the environment and address floods (WFERF).

## Information and methods

### Research area

**FIGURE 2:**
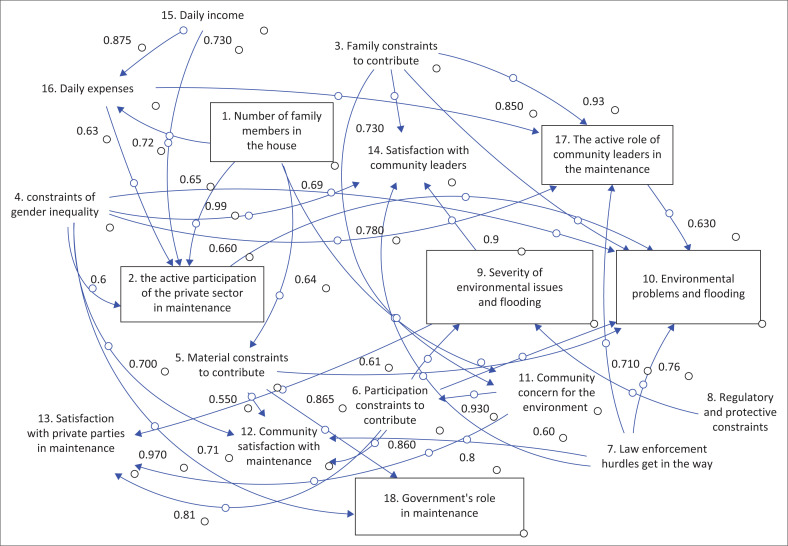
Stock diagram of Surabaya City.

This research was conducted in three major cities in East Java province, Indonesia, which regularly contend with annual floods during the rainy season. These cities include Surabaya, Sidoarjo and Malang, as shown in Figure 2. Surabaya, the provincial capital and the largest metropolitan centre in East Java, is the second-largest city in Indonesia after Jakarta. Geographically, Surabaya is located 800 km east of Jakarta or 435 km northwest of Denpasar, Bali. It occupies a strategic location along the northern coast of the eastern part of Java Island, overlooking both the Madura Strait and the Java Sea. The city spans an area of approximately ± 326.81 km^2^ and had a population of 2 970 843 as of 30 June 2021. Sidoarjo played a significant role as a supporting city for Surabaya and had a population of 2 266 533 in 2019. It is geographically situated between 112°5’ and 112°9’ East Longitude as well as 7°3’ and 7°5’ South Latitude. Malang, the second-largest city within East Java, occupies a highland terrain covering 145.28 km^2^ and is home to a population of 895 387. It is also renowned as a city of education.

### Methodology

This study uses the methodology of survey questionnaires and interviews. In line with developing the community participation model for environmental conservation and flood management, the survey questionnaire comprises 13 distinct sections. Sections 1–3 were dedicated to matters at the household level, while Section 4 gathers demographic information about family members. Section 5 involves interviews to delve into environmental management programmes and associated challenges, followed by Section 6, which centres on assessing the influence of governmental bodies and local figures concerning environmental conservation and flood incidents. Subsequently, Section 7 is designed to evaluate material aspects of the living environment. Section 8 focuses on uncovering satisfaction levels and roles within endeavours for environmental conservation. Transitioning to Section 9, the assessment pertains to the extent of concern regarding environmental issues within the vicinity. Section 10 delves into the role of women in environmental and flood-related concerns, while Section 11 evaluates material incentives about environmental concerns. Section 12 was dedicated to understanding the hurdles that hinder contributions to environmental and flood-related causes. Lastly, Section 13 captures the aspirations of women concerning issues related to the environment and flood-related issues.

### Data collection

Instrument for data collection during the COVID-19 pandemic involved utilising two methods, including employing a Google Form survey questionnaire and conducting face-to-face interviews. The face-to-face interviews were conducted with strict adherence to health protocols mandated by local authorities, specifically during the implementation of Emergency Situation 2 in the city from 06 July to 06 August 2021. The research team distributed the questionnaire with valuable support from undergraduate Civil Engineering students who possessed fundamental knowledge about environmental and flood-related issues and expertise in questionnaire collection. The leader played a supervisory role throughout the distribution process, ensuring the validity of the collected data, which adhered to the principle of voluntariness. Respondents were not obligated to complete the questionnaire sequentially, a measure designed to prevent data distortion. Furthermore, they had full autonomy in choosing whether to participate or withdraw from the survey. As a gesture of appreciation for their involvement, each respondent received a token of remembrance upon completing the questionnaire. A total number of 71, 74 and 61 respondents were found in Malang, Sidoarjo City, and Surabaya.

### Statistical analysis method

The model development incorporates a set of 18 different variables analysed in three statistical stages. The first stage entails descriptive statistics, a vital step for summarising the characteristics of the data and gaining insights into its basic distribution. The second stage necessitates correlation analysis to discern whether the hypothesised influencing factors significantly impact the variables. The correlation coefficients of these influencing factors were calculated using Equation 1:

$$r=\frac{\text{n}\Sigma \text{xy}-(\Sigma \text{y})}{\surd \{\text{n}\Sigma {\text{x}}^{2}-(\Sigma {\text{x}}^{2})\}\{\text{n}\Sigma {\text{y}}^{2}-{\left(\Sigma \text{y}\right)}^{2}\}}$$ [Eqn 1]

where:

n = Number of X and Y data pairs

Σx = Total sum of variable X

Σy = Total sum of Y variables

Σx^2^ = Square of total number of variables X

Σy^2^ = Square of total number of variables Y

Σxy = The multiplication result of the total X and Y variables number.

The correlation threshold is set at a minimum of *r* = 0.6, indicating the lower limit for acceptable correlation. Meanwhile, variables exhibiting a correlation value below 0.6 were excluded because of their weaker association with other variables. The subsequent stage involves the construction of a causal loop diagram, succeeded by developing a stock-flow diagram. The causal loop diagram comprises interconnected variables linked by arrows, visually depicting their cause-and-effect connections (Proust & Newell 2020). The model development is undertaken to ascertain behavioural patterns and the interplay among variables within the simulation. This evaluation determines the aptness of the model to replicate real-world behaviours and interactions (Tables 1, 2, 3, and 4). Table 1 is the variables used in the research. Table 2 is the Relationship between factors impacting the environment and flooding in Sidoarjo city, Table 3 is the Relationship between factors impacting the environment and flooding in Surabaya City and Table 4 is the Relationship between factors impacting the environment and flooding in Malang City.

**TABLE 1:** Research variables.

Number	English	Abbreviations
1.	Number of family members in the household	NOFMITH
2.	The activities of the private sector in maintenance	TAOTPSIM
3.	Family constraints to contribute	FCTC
4.	Gender inequality barriers	GIB
5.	Material constraints to contribute	MCTC
6.	Participation barriers to contribute	PBTC
7.	Obstruction of law enforcement	OOLE
8.	Regulatory and protection constraints	RAPC
9.	The severity of environmental issues and flooding in the city	TSOEIAFITC
10.	The severity of environmental problems and flooding in the city	TSOEPAFITC
11.	Public concern for the environment and flooding in the city	PCFTEAFITC
12.	Community satisfaction with the government	PSWTG
13.	Satisfaction with the private sector and the government	SWTPSATG
14.	Satisfaction with community leaders	SWL
15.	Daily income	DI
16.	Daily expenses	DE
17.	The active role of community leaders in the maintenance	AOCLIM
18.	The role of government in maintenance	TROGIM
19.	Family problems get in the way	FPGITW
20.	Material constraints hinder contributions	MCHC
21.	Participation barriers get in the way	PBGITW
22.	Obstacles to law enforcement	OTLE
23.	Disruption of environmental issues and flooding in the city	DOEIAFITC
24.	Number of household members	NOHM
25.	The active role of the private sector	TAROTPC
26.	Concern for the environment	CFTE
27.	Satisfaction of the private sector in government policy	SOTPSI
28.	Satisfaction with government	SWG
29.	Active role of community leaders	AROCL

**TABLE 2:** Relationship between factors impacting the environment and flooding in Sidoarjo city.

Variable	1	2	3	4	5	6	7	8	9	10	11	12	13	14	15	16	17	18
NOFMITH	-	-	-	-	-	-	-	-	-	-	-	-	-	-	-	-	-	-
TAOTPSIM	0.14	-	-	-	-	-	-	-	-	-	-	--	-	-	-	-	-	-
FCTC	0.65	0.57	-	-	-	-	-	-	-	-	-	-	-	-	-	-	-	-
GIB	0.62	0.66	< 0.001	-	-	-	-	-	-	-	-	-	-	-	-	-	-	-
MCTC	0.11	0.83	0.00	0.00	-	-	-	-	-	-	-	-	-	-	-	-	-	-
PBTC	0.83	0.67	0.02	0.01	< 0.001	-	-	-	-	-	-	-	-	-	-	-	-	-
OOLE	0.03	0.97	0.06	0.16	0.00	0.00	-	-	-	-	-	-	-	-	-	-	-	-
RAPC	0.07	0.59	0.01	0.11	< 0.001	< 0.001	< 0.001	-	-	-	-	-	-	-	-	-	-	-
TSOEIAFITC	0.35	0.98	0.12	0.04	< 0.001	0.01	0.00	0.00	-	-	-	-	-	-	-	-	-	-
TSOEPAFITC	0.53	0.50	0.14	0.02	< 0.001	0.03	0.02	0.00	< 0.001	-	-	-	-	-				
PCFTEAFITC	0.57	0.98	0.37	0.56	0.43	0.94	0.83	0.73	0.28	0.80	-	-	-	-	-	-	-	-
PSWTG	0.36	0.03	0.47	0.87	0.56	0.31	0.39	0.37	0.34	0.37	0.04	-	-	-	-	-		
SWTPSATG	0.17	< 0.001	0.91	0.76	0.50	0.40	0.77	0.52	0.73	0.83	0.85	0.07	-	-	-	-	-	-
SWL	0.02	0.26	0.65	0.22	0.94	0.75	0.15	0.26	0.40	0.46	0.00	0.02	0.33	-	-	-	-	-
DI	0.91	0.54	0.25	0.27	0.60	0.68	0.16	0.73	0.80	0.41	0.24	0.58	0.52	0.83	-	-	-	-
DE	0.73	0.75	0.54	0.24	0.87	0.65	0.14	0.77	0.62	0.25	0.23	0.46	0.30	0.94	< 0.001	-	-	-
AOCLIM	< 0.001	0.05	0.76	0.43	0.97	0.78	0.19	0.24	1.00	0.67	0.03	0.00	0.03	< 0.001	0.69	0.59	-	-
TROGIM	0.70	0.12	0.33	0.87	0.72	0.92	0.46	0.24	0.58	0.37	0.02	< 0.001	0.39	0.01	0.71	0.41	0.24	-

NOFMITH, Number of family members in the household; TAOTPSIM, The activities of the private sector in maintenance; FCTC, Family constraints to contribute; GIB, Gender inequality barriers; MCTC, Material constraints to contribute; PBTC, Participation barriers to contribute; OOLE, Obstruction of law enforcement; RAPC, Regulatory and protection constraints; TSOEIAFITC, The severity of environmental issues and flooding in the city; TSOEPAFITC, The severity of environmental problems and flooding in the city; PCFTEAFITC, Public concern for the environment and flooding in the city; PSWTG, Community satisfaction with the government; SWTPSATG, Satisfaction with the private sector and the government; SWL, Satisfaction with community leaders; DI, Daily income; DE, Daily expenses; AOCLIM, The active role of community leaders in the maintenance; TROGIM, The role of government in maintenance.

**TABLE 3:** Relationship between factors impacting the environment and flooding in Surabaya City.

Variable	1	2	3	4	5	6	7	8	9	10	11	12	13	14	15	16	17	18
NOFMITH	-	-	-	-	-	-	-	-	-	-	-	-	-	-	-	-	-	-
TAOTPSIM	0.659	-	-	-	-	-	-	-	-	-	-	-	-	-	-	-	-	-
FPGITW	0.325	0.318	-	-	-	-	-	-	-	-	-	-	-	-	-	-	-	-
GIB	0.342	0.643	< 0.001	-	-	-	-	-	-	-	-	-	-	-	-	-	-	-
MCHC	0.636	0.303	0.007	< 0.001	-	-	-	-	-	-	-	-	-	-	-	-	-	-
PBGITW	0.557	0.574	0.034	0.405	0.149	-	-	-	-	-	-	-	-	-	-	-	-	-
OTLE	0.369	0.164	< 0.001	< 0.001	0.014	0.024	-	-	-	-	-	-	-	-	-	-	-	-
RAPC	0.152	0.386	< 0.001	< 0.001	< 0.001	0.278	< 0.001	-	-	-	-	-	-	-	-	-	-	-
TSOEIAFITC	0.092	0.583	0.020	0.173	0.206	0.783	0.037	0.141	-	-	-	-	-	-	-	-	-	-
TSOEPAFITC	0.295	0.645	0.659	0.737	0.604	0.672	0.746	0.622	0.002	-	-	-	-	-	-	-	-	-
PCFTEAFITC	0.690	0.525	0.644	0.022	0.074	0.210	0.698	0.010	0.540	0.448	-	-	-	-	-	-	-	-
PSWTG	0.039	0.009	0.806	0.703	0.654	0.865	0.602	0.430	0.182	0.179	0.851	-	-	-	-	-	-	-
SWTPSATG	0.509	< 0.001	0.453	0.299	0.091	0.807	0.253	0.138	0.975	0.462	0.873	0.006	-	-	-	-	-	-
SWL	0.048	0.049	0.739	0.990	0.128	0.400	0.931	0.504	0.971	0.492	0.451	0.002	0.026	-	-	-	-	-
DI	0.370	0.727	0.366	0.482	0.051	0.418	0.302	0.620	0.701	0.828	0.998	0.964	0.660	0.112	-	-	-	-
DE	0.723	0.639	0.220	0.350	0.356	0.217	0.217	0.238	0.574	0.215	0.082	0.944	0.570	0.998	0.874	-	-	-
AOCLIM	0.046	0.051	0.930	0.781	0.112	0.302	0.702	0.327	0.700	0.645	0.574	0.006	0.026	< 0.001	0.178	0.859	-	-
TROGIM	0.014	0.389	0.254	0.699	0.851	0.259	0.334	0.487	0.070	0.115	0.435	< 0.001	0.424	0.007	0.620	0.415	0.009	-

NOFMITH, Number of family members in the household; TAOTPSIM, The activities of the private sector in maintenance; FPGITW, Family problems get in the way; GIB, Gender inequality barriers; MCHC, Material constraints hinder contributions; PBGITW, Participation barriers get in the way; OTLE, Obstacles to law enforcement; RAPC, Regulatory and protection constraints; TSOEIAFITC, The severity of environmental issues and flooding in the city; TSOEPAFITC, The severity of environmental problems and flooding in the city; PCFTEAFITC, Public concern for the environment and flooding in the city; PSWTG, Community satisfaction with the government; SWTPSATG, Satisfaction with the private sector and the government; SWL, Satisfaction with community leaders; DI, Daily income; DE, Daily expenses; AOCLIM, The active role of community leaders in the maintenance; TROGIM, The role of government in maintenance.

**TABLE 4:** Relationship between factors impacting the environment and flooding in Malang City.

Variable	1	2	3	4	5	6	7	8	9	10	11	12	13	14	15	16	17	18
DOEIAFITC	-	-	-	-	-	-	-	-	-	-	-	-	-	-	-	-	-	-
NOHM	0.691	-	-	-	-	-	-	-	-	-	-	-	-	-	-	-	-	-
TAROTPC	0.928	0.380	-	-	-	-	-	-	-	-	-	-	-	-	-	-	-	-
FPGITW	0.500	0.311	0.144	-	-	-	-	-	-	-	-	-	-	-	-	-	-	-
GIB	0.112	0.902	0.668	0.142	-	-	-	-	-	-	-	-	-	-	-	-	-	-
MCTC	0.726	0.986	0.605	0.434	0.507	-	-	-	-	-	-	-	-	-	-	-	-	-
PBTC	0.209	0.210	0.118	0.101	0.880	0.571	-	-	-	-	-	-	-	-	-	-	-	-
OTLE	0.031	0.751	0.569	0.136	< 0.001	0.715	0.980	-	-	-	-	-	-	-	-	-	-	-
RAPC	0.006	0.402	0.440	0.066	0.004	0.745	0.331	< 0.001	-	-	-	-	-	-	-	-	-	-
TSOEPAFITC	0.008	0.440	0.630	0.913	0.840	0.187	0.164	0.645	0.274	-	-	-	-	-	-	-	-	-
PCFTEAFITC	0.986	0.340	0.379	0.213	0.343	0.113	0.740	0.567	0.740	0.936	-	-	-	-	-	-	-	-
SOTPSI	0.691	0.547	< 0.001	0.116	0.618	0.565	0.082	0.500	0.353	0.615	0.609	-	-	-	-	-	-	-
SWG	0.570	0.503	0.082	0.207	0.169	0.237	0.112	0.393	0.479	0.730	0.877	0.052	-	-	-	-	-	-
SWL	0.318	0.081	0.600	0.851	0.664	0.884	0.825	0.782	0.542	0.371	0.169	0.460	0.163	-	-	-	-	-
DI	0.326	0.232	0.671	0.659	0.440	0.709	0.206	0.815	0.433	0.493	0.458	0.740	0.819	0.459	-	-	-	-
DE	0.365	0.495	0.343	0.426	0.316	0.945	0.205	0.632	0.281	0.665	0.703	0.445	0.610	0.884	< 0.001	-	-	-
AROCL	0.275	0.140	0.412	0.957	0.677	0.849	0.773	0.865	0.612	0.260	0.210	0.279	0.207	< 0.001	0.420	0.872	-	-
TROGIM	0.439	0.795	0.021	0.221	0.486	0.612	0.009	0.734	0.522	0.822	0.920	0.007	< 0.001	0.187	0.540	0.330	0.210	-

DOEIAFITC, Disruption of environmental issues and flooding in the city; NOHM, Number of household members; TAROTPC, The active role of the private sector; FPGITW, Family problems get in the way; GIB, Gender inequality barriers; MCTC, Material constraints to contribute; PBTC, Participation barriers to contribute; OTLE, Obstacles to law enforcement; RAPC, Regulatory and protection constraints; TSOEPAFITC, The severity of environmental problems and flooding in the city; PCFTEAFITC, Public concern for the environment and flooding in the city; SOTPSI, Private sector satisfaction in government policy; SWG, Satisfaction with government; SWL, Satisfaction with community leaders; DI, Daily income; DE, Daily expenses; AROCL, Active role of community leaders; TROGIM, The role of government in maintenance.

### Findings

Government policy represents actions initiated and enforced by authorised government entities, encompassing legal, political and financial authorities. An effective approach the government can employ to address environmental issues involves implementing relevant laws and imposing stringent sanctions on individuals or entities that intentionally or unintentionally contravene these regulations (Sholihah et al. 2020). In accordance with the research findings, it becomes evident that the government plays a crucial role in maintaining the environment and managing flooding issues in East Java province, Indonesia.

### Disaster education

The impacts of flooding consequences extend to the quality of raw water, which can be negatively affected by contamination. Meanwhile, the impact of other environmental issues, such as air and water pollution, is caused by many irresponsible parties committing violations by building factories on the banks of the river. This can lead to river water overflowing, causing damage to buildings, embankments, settlements, among others (Sholihah et al. 2020), and healthcare volunteers are useful for coordinating and helping those who are impacted.

### Disaster preparedness

Effective environmental maintenance necessitates implementing well-structured regional strategies that involve the active participation of various stakeholders, including universities, the private sector, NGOs, among others. These stakeholders can significantly contribute to reinforcing empowerment programmes. According to Coglianese and Nash (2010), government policymakers have not been the only ones to target firms’ internal management in an attempt to improve private sector environmental performance.

### Disaster information

The impact of urban floods on the lives of the urban population, particularly their family members’ income, is significant. A similar situation was observed in Bangkok, where losses amounted to approximately half of the annual household expenditure (Nabangchang et al. 2015). Government compensation payments did little to alleviate the overall economic losses experienced by most households (Cao et al. 2020). Meanwhile, because of flooding, many families are not insured and do not have sufficient savings to rebuild, and government assistance is limited (Netusil et al. 2021). In East Java, it is apparent that income and the number of family members in households substantially influence concerns related to the environment and flood hazards. This is reinforced by the fact that the community’s income, in general, has experienced a significant decline during the pandemic.

## Discussion

**FIGURE 3:**
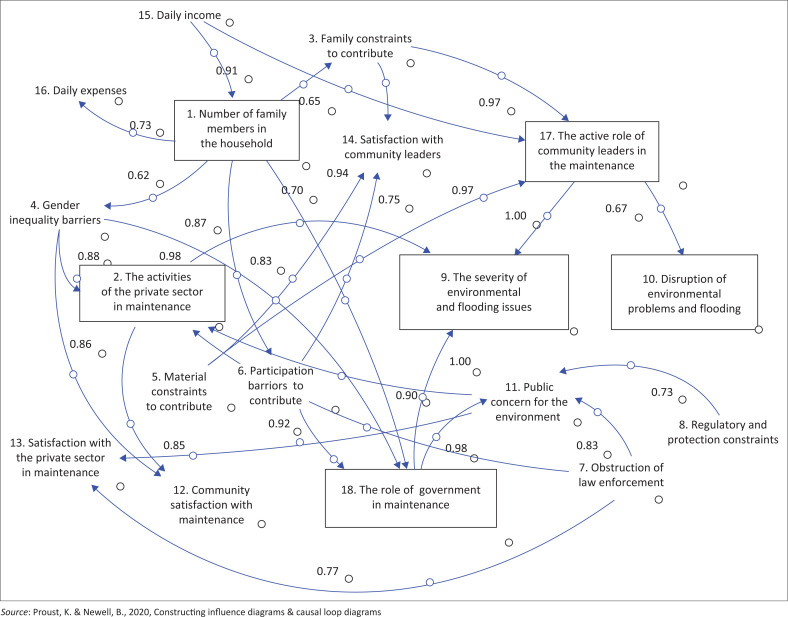
Stock diagram of Sidoarjo city.

**FIGURE 4:**
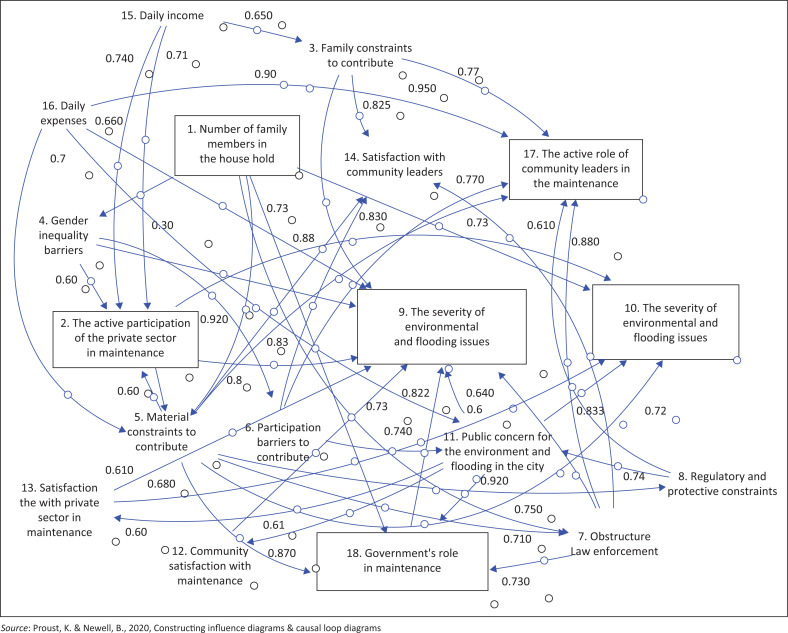
Stock diagram of Malang City.

The outcomes generated by the Vensim program illustrate the model development for Surabaya City, as shown in Figure 2. Similarly, Figure 3 represents the model for Sidoarjo City, while Figure 4 shows the model for Malang City.

**FIGURE 5:**
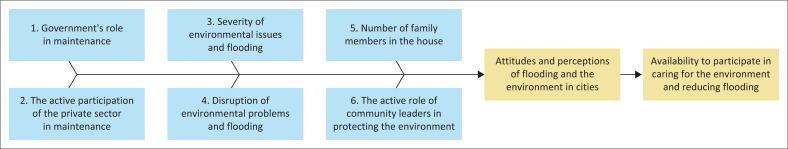
Model to increase community willingness to care for the environment and reduce flooding.

The data analysis of the stock diagram results shows that the triangle theory development model becomes the WFERF Model divided into six components and is very influential in East Java province. These components include the role of the government in maintaining the environment that causes flooding, the activeness of the private sector, the severity of environmental and flooding issues, disruption, the number of family members, and the active role and existence of community leaders in protecting the environment as shown in Figure 5.

### Hypothesis 1: The government’s role in environmental maintenance

Figure 4 shows that the people of Malang City have contributed less to the flood environment than the cities of Surabaya and Sidoarjo. This is influenced by community contributions and participation (0.92), gender equality (0.87), and the number of families (0.79).

### Hypothesis 2: Private sector involvement in environmental maintenance

Figure 2 illustrates how the private sector’s involvement in environmental maintenance has a substantial impact on public satisfaction with environmental maintenance (0.85) and the seriousness of environmental and flooding issues (0.96). However, the private sector faces certain obstacles, including a lack of law enforcement response (0.96) and insufficient public concern (0.98). In Figure 3, the activeness of the private sector in maintenance is impacted by the daily income (0.74) of the community and contributing material (0.6). In Figure 2, which pertains to Surabaya, a major Indonesian city with a thriving private sector because of numerous factories and companies, the role of this sector is heavily influenced by economic factors, including the number of family members (0.99) and daily income (0.73).

### Hypothesis 3: Severity of environmental and flooding issues

Handayani et al. (2020) stated that the severity of environmental and flooding issues is often attributed to changes in land use around watershed areas, particularly in urban areas where green open spaces are converted into housing. Therefore, policies related to land use are needed. According to Chiang (2018), flooding can be exacerbated by heavy rainfall because of global climate change, potentially leading to flash floods in flood-prone urban areas and endangering the lives and safety of residents. In the city of Sidoarjo according to Figure 3 The active role of community leaders in the maintenance and The role of government in maintenance have the same influence on the conditions of The severity of environmental and flooding issues. The severity of environmental and flooding issues in Malang City is impacted by the role of the government in environmental maintenance activities (0.822) and community satisfaction with environmental maintenance (073). This is in addition to the family constraints contributing to environmental and flooding issues (0.69) and satisfaction with the private sector (0.6), as shown in Figure 4. The activeness of the private sector is only small (0.63) because Malang City is a city of education. However, public concern for the environment is very large (0.92).

### Hypothesis 4: Disruption of environmental and flooding issues

In Malang City, flooding is impacted by community concern (0.94) and the activeness of the private sector in environmental maintenance (0.88), material constraints to contribute (0.72), number of members in the house (0.78) as well as satisfaction with the private sector (0.74).

### Hypothesis 5: Number of family members in the house

Urban floods have a substantial effect on the lives of those who reside there, especially on the financial status of their families. A comparable circumstance was noted in Bangkok, where losses were almost equal to half of the annual household budget (Nabangchang et al. 2015). According to Cao et al. (2020), the majority of households suffered overall economic losses that were not significantly mitigated by government compensation payments. In the meanwhile, there is little government support due to flooding, and many households lack the insurance and money necessary to reconstruct (Netusil et al. 2021). Regrettably, a lot of flood-affected households don’t have enough money saved for reconstruction or insurance, and government help is sometimes scarce. It is evident in East Java that worries are significantly influenced by household wealth and the number of family members.

This is reinforced by the fact that the community’s income, in general, has experienced a significant decline during the pandemic. Figure 3 shows that in Sidoarjo city, the number of family members in the house is strongly impacted by daily income (0.91) and impacts participation constraints to contribute (0.83), daily expenditure, the number of responses to the government’s role in maintenance (0.7) and gender equality constraints (0.62). Figure 2 shows that in Surabaya City, the number of family members in the house impacts daily expenditure (0.72), the activity of the private sector in maintenance (0.99) and community concern for the environment and flooding (0.69). Then, Figure 4 shows that the number of family members in the house greatly impacts the material or financial family; therefore, the contribution to environmental and flooding concerns is less.

### Hypothesis 6: The active role of community leaders in protecting the environment

Community leaders play a crucial role in safeguarding the environment, particularly during challenging times like the pandemic, where their concern revolves around the rapid spread of disease and its adverse societal impact. In response, these leaders use preventive measures to protect the environment and invite community members to join. They campaigned for healthy living behaviours and initiated actions aimed at helping community members whose socio-economic conditions were devastated by the pandemic. This proactive stance demonstrates the significant role that community leaders play in maintaining a healthy environment within their communities during such crises (Rosidin, Rahayuwati & Herawati 2020). Many community leaders support the government’s environmental policies, but when unprofessional leadership is involved in politicised decision-making, it fails to enforce the provisions of the law. In addition, Figure 3 shows that in Sidoarjo city, the active role of community leaders in protecting the environment greatly impacts the severity of environmental and flooding issues (Mashiane et al. 2023) and the disruption of environmental issues and flooding (0.67). Figure 2 shows that the severity of environmental and flooding issues in Surabaya City is caused by regulatory constraints (0.71) and significantly impacts the satisfaction of community leaders (0.9). Moreover, Figure 4 shows that in Malang City, the active role of community leaders in protecting the environment is strongly impacted by law enforcement (0.86) and existing regulations.

## Conclusion

Community opinions and attitudes towards the environment and flooding issues in cities were impacted by watershed management and environmental maintenance in cities, specifically during the pandemic. Therefore, to determine the factors that impacted the willingness of the community to participate in protecting the environment and dealing with floods, the triangular relationship development method, namely WFERF, was used. This method was appropriately applied in urban areas and was adapted by countries like Indonesia. Appropriate to the results and analysis of research data using a correlation value of at least *r* = 0.6 and a minimum correlation level, the result showed that six dominant factors in urban communities impacted the state of the environment and flooding issues in the city. Environmental problems, especially flooding, in East Java province are more influenced by the number of people in the household and the role of community leaders with a correlation value of 0.62 < *r* < 0.99. In general, during the pandemic, people in East Java Province are very aware of the importance of maintaining a clean and comfortable environment. In general, during the pandemic, the community in the East Java province was very aware of the importance of maintaining environmental cleanliness and comfort.
